# Bicuspid Aortic Valve Endocarditis Caused by Streptococcus sanguinis: A Case Report

**DOI:** 10.7759/cureus.52488

**Published:** 2024-01-18

**Authors:** Sravani Singu, Robert Chory, Vamsi K Singu, Michael Pursley, Glenn Harris

**Affiliations:** 1 Internal Medicine, Thomas Hospital, Infirmary Health, Fairhope, USA; 2 Neuroscience, Thomas Hospital, Infirmary Health, Fairhope, USA; 3 Neuroscience, University of Alabama at Birmingham, Birmingham, USA; 4 Cardiovascular Disease, Thomas Hospital, Infirmary Health, Fairhope, USA

**Keywords:** aortic valve insufficiency, aortic insufficiency, prophylactic antibiotics, dental procedure, aortic regurgitation, infective endocarditis, bicuspid aortic valve, streptococcus sanguinis

## Abstract

Antibiotic prophylaxis prior to dental work in bicuspid aortic valve (BAV) patients is currently a matter of debate. The American Dental Association does not require those with native BAV to receive antibiotic prophylaxis prior to dental work as BAV is considered an “intermediate” risk for infective endocarditis (IE). We present the case of a 63-year-old male, with a medical history of BAV, who acquired* Streptococcus sanguinis* IE after a routine dental cleaning four months prior to initial onset of symptoms. He exhibited new-onset and severe aortic regurgitation at presentation, requiring urgent aortic valve replacement to restore valve function. BAV patients are at high risk of IE, emphasizing the need for prophylactic antibiotics in dental cleaning as well as invasive dental procedures in those with BAV.

## Introduction

Infective endocarditis (IE) is characterized by inflammation of the inner lining of the heart, the endocardium, and any of the four heart valves, most commonly the tricuspid valve. The incidence of IE is approximately seven per 100,000 people per year and carries up to a 30% mortality risk. The most common causative pathogens of IE are gram-positive *Streptococci*, *Staphylococci*, and *Enterococci* as they are normal colonizers of the oral cavity [1]. Of all cases, 80-90% are caused by one of the three groups of pathogens. Less frequent culprits of IE include the HACEK organisms (*Haemophilus*, *Aggregatibacter*, *Cardiobacterium*, *Eikenella*, and *Kingella*), which are normal colonizers of the oropharynx [1]. Bicuspid aortic valve (BAV) has a prevalence of 0.5-2% and is the most common congenital cardiac abnormality [2]. The incidence of native valve IE in those with BAV is 2-5% and is most commonly seen in younger male patients according to the literature [1]. The pathophysiology of IE in BAV is hypothesized to be due to endothelial damage caused by abnormal shear stress from the turbulent flow of blood across bicuspid valves. Endothelial damage leads to platelet and fibrinogen deposition, which leads to fibrosis and valve calcification, which becomes a nidus for bacterial or fungal seeding [2]. Here we present a unique case of a 63-year-old male, with a medical history of BAV, who acquired *Streptococcus sanguinis* IE thought to be triggered by routine dental cleaning four months prior to onset of symptoms and severe aortic insufficiency. Early identification and intervention are important in preventing complications and death.

## Case presentation

**Figure 1:**
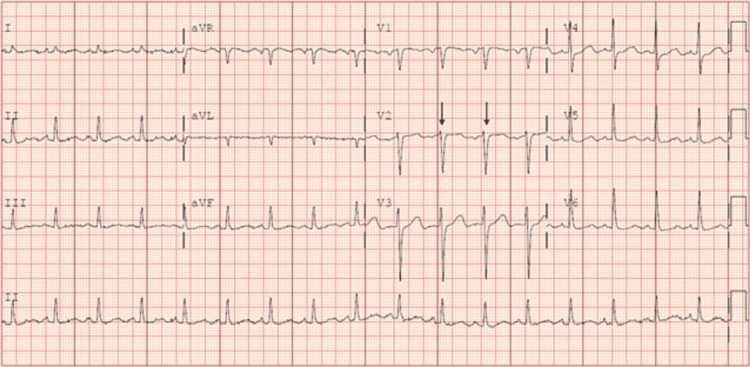
Electrocardiogram showing sinus tachycardia with arrows pointing out RR interval

**Figure 2:**
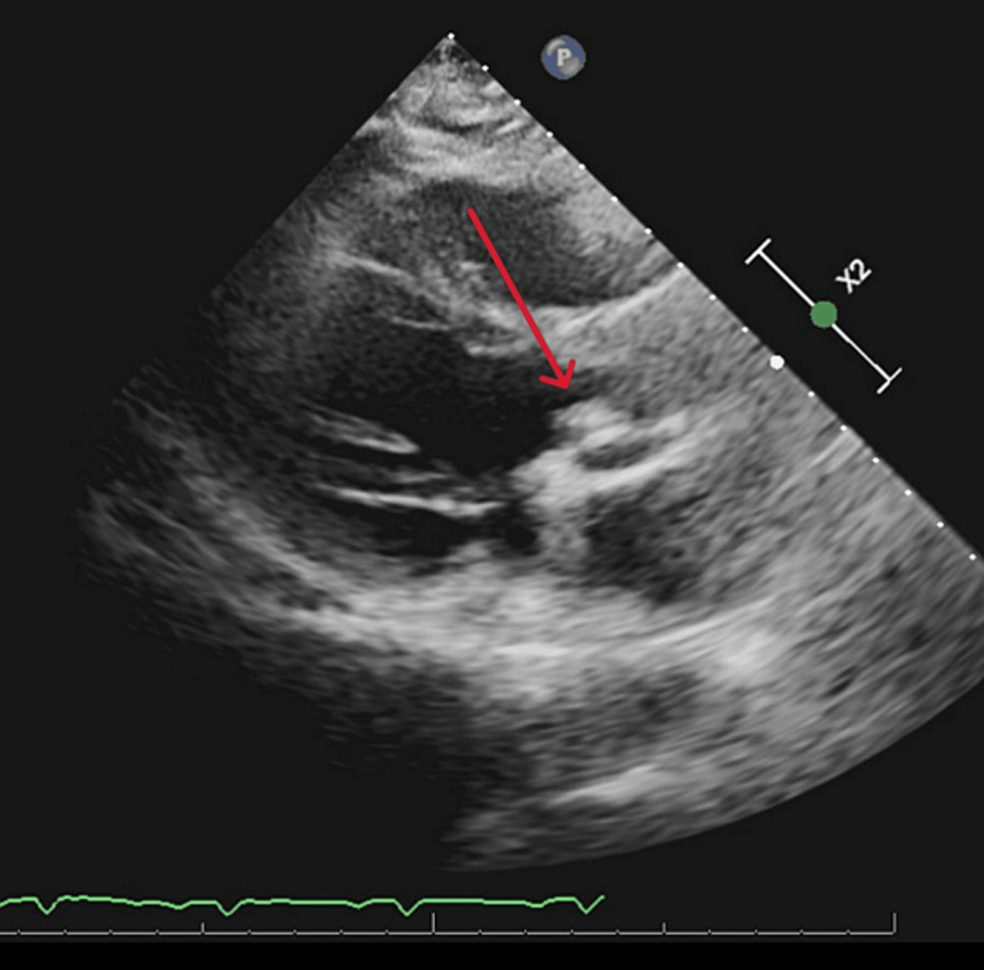
BAV vegetation on transthoracic echocardiogram BAV: bicuspid aortic valve

**Figure 3:**
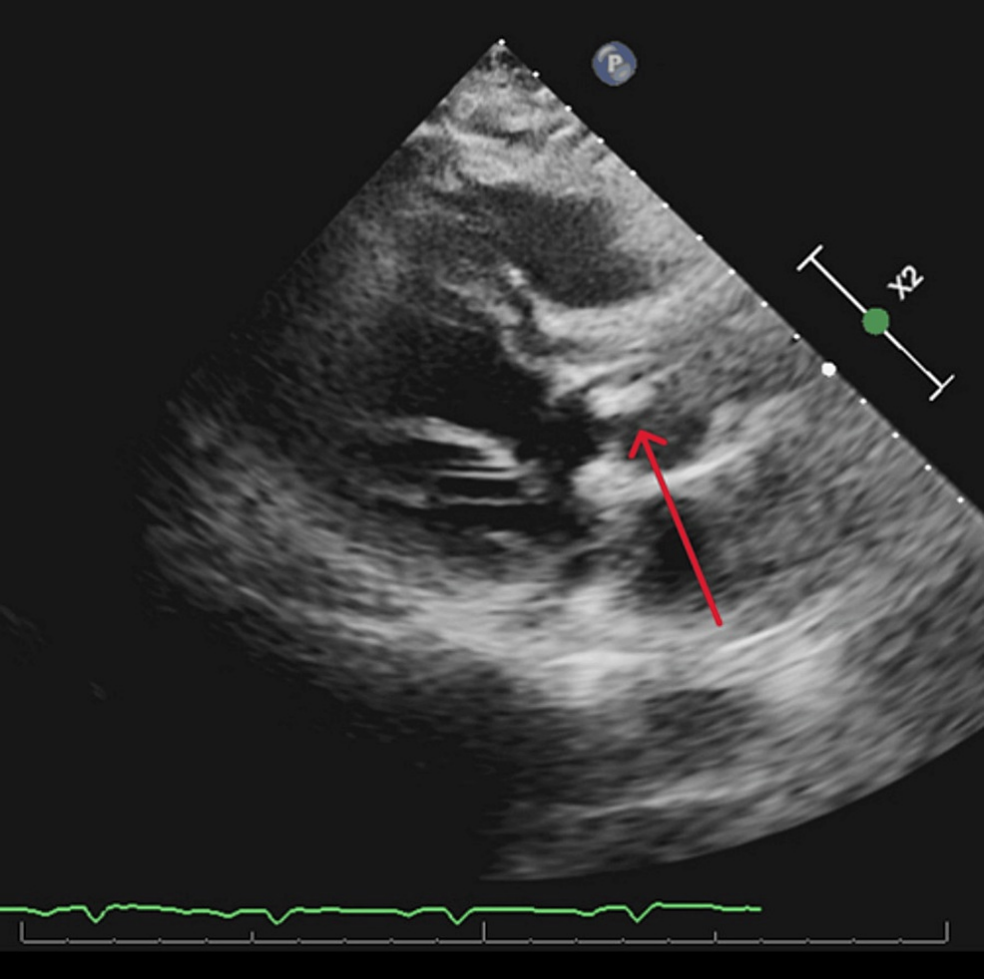
BAV vegetation on transthoracic echocardiogram BAV: bicuspid aortic valve

**Figure 4:**
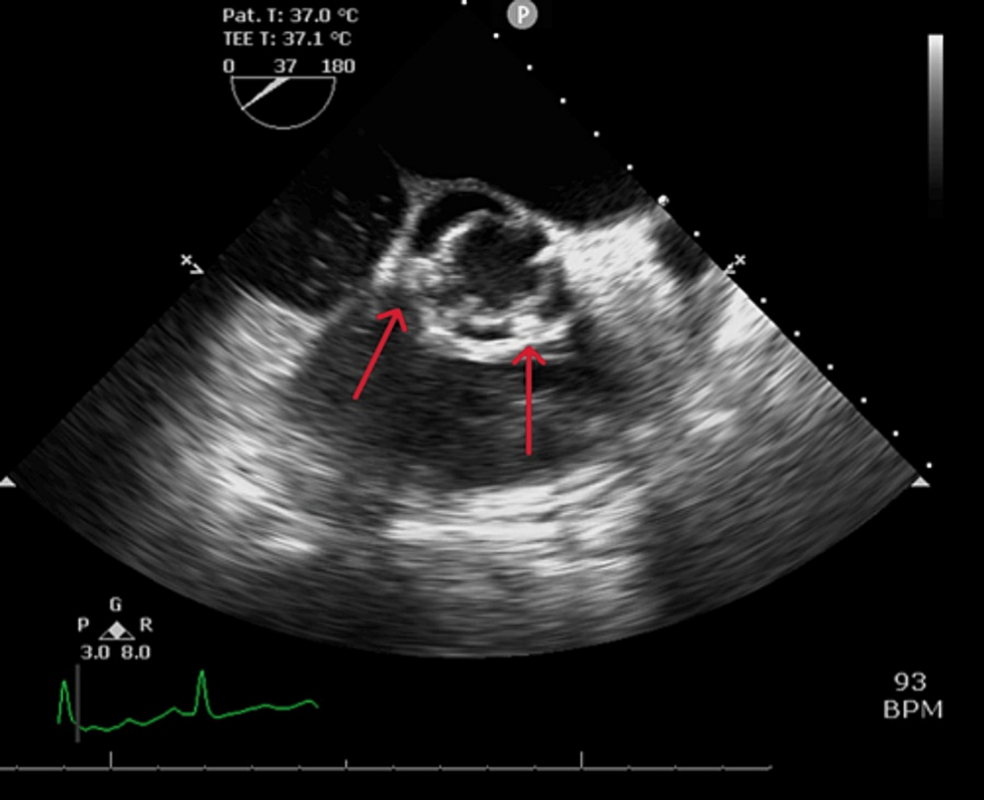
BAV vegetations on transesophageal echocardiogram BAV: bicuspid aortic valve

**Figure 5:**
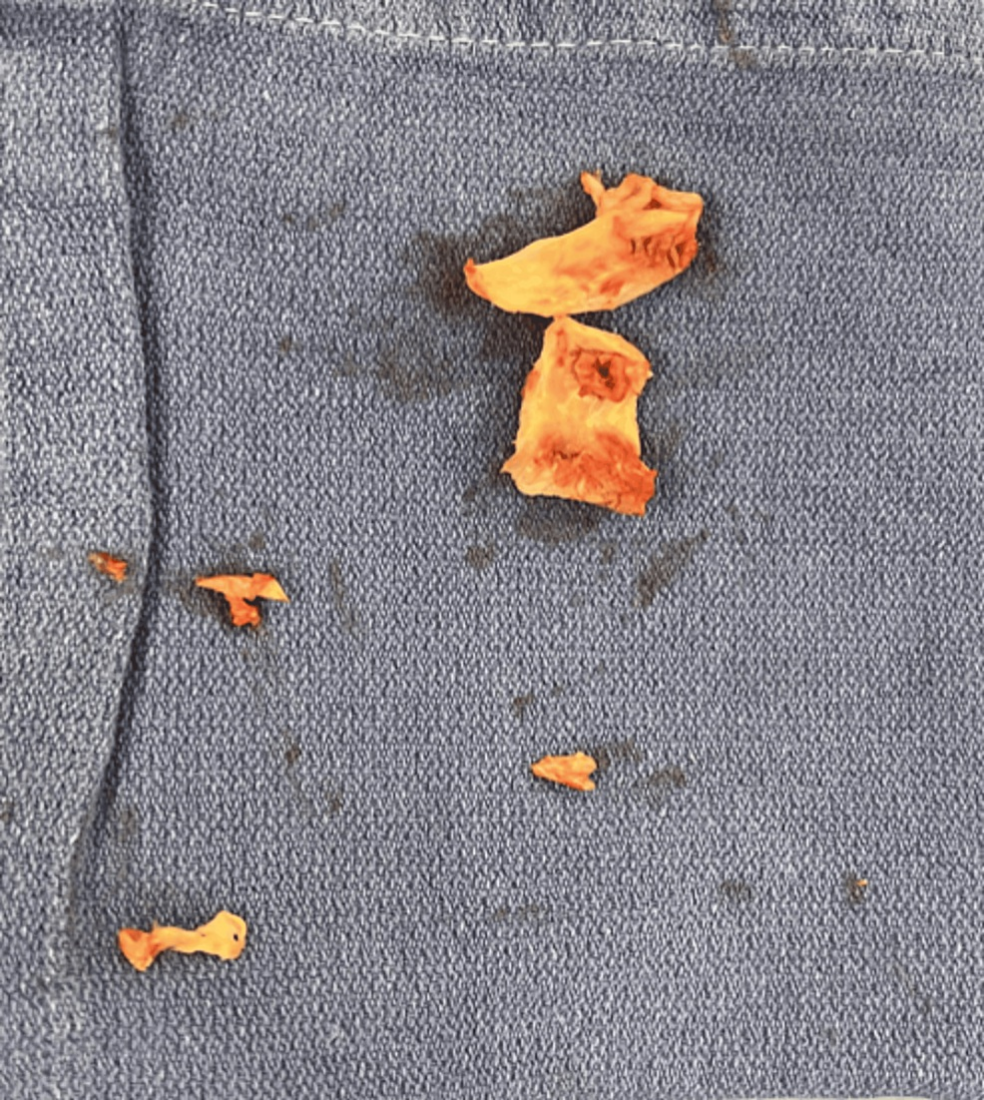
Aortic valve remnant

A 63-year-old male with a medical history of BAV and hypertension presented to our ED with a two-week history of fevers, generalized fatigue, and dyspnea on exertion. Review of systems was positive for poor appetite, 20-pound weight loss over six weeks, and night sweats. He underwent a routine dental cleaning four months prior to onset of symptoms. His medical history is significant for BAV. Vital signs on presentation were blood pressure 112/62 mmHg, heart rate 106 beats per minute, respiratory rate 20 breaths per minute, temperature 97.8 degrees Fahrenheit, and saturation 93% on room air. WBC count was elevated at 13 k/μL, hemoglobin was 10 g/dL with a baseline of around 12 g/dL, electrolytes were within normal limits, and creatinine was elevated at 1.4 mg/dL with a baseline of around 1.0 mg/dL. On physical examination, the patient was not in acute distress, had a 2 out of 6 diastolic heart murmur over the aortic and pulmonic areas, was tachycardic with a heart rate in the 100s (Figure 1), and had reduced breath sounds over bilateral lung bases. Oral exam revealed good dental hygiene. All other systems were unremarkable on the physical exam. Electrocardiography showed sinus tachycardia with probable left atrial enlargement. Chest x-ray revealed right greater than left mild pleural effusions and atelectasis in lower lung lobes. Transthoracic echocardiogram showed normal left ventricular ejection fraction, severe aortic regurgitation, and moderate-sized vegetation or mass on the BAV (Figures 2, 3). A transesophageal echocardiogram confirmed vegetations on the ventricular surfaces of both aortic leaflets and severe aortic regurgitation (Figure 4). Empiric antibiotic therapy was initiated with intravenous vancomycin and ceftriaxone. After 28 hours of incubation, two sets of blood cultures grew gram-positive cocci in chains and a Biofire PCR (bioMérieux SA, Marcy-l'Étoile, France) blood culture panel was positive for S. *sanguinis*, sensitive to both vancomycin and Rocephin. The antibiotic choice was narrowed down to IV ceftriaxone based on sensitivities. The patient underwent surgical aortic valve replacement with a mechanical valve without complications. Figure 5 shows the remnant BAV with vegetation. He was started on warfarin with daily international normalized ratio (INR) monitoring inpatient until it was within the therapeutic range between two and three. The explanted aortic valve was cultured and grew alpha-hemolytic *Streptococcus*. He also had repeat blood cultures drawn that did not yield bacterial growth and was discharged on intravenous ceftriaxone for eight weeks. The patient has since followed up in the cardiology clinic and has been doing well.

## Discussion

The initial Duke criteria were published in 1994 and modified in 2000. An updated set of criteria has recently been proposed by the International Society for Cardiovascular Infectious Disease (ISCVID) [3]. The new criteria were developed primarily for the evaluation of left-sided native valve IE [3]. The sensitivity of the criteria is decreased in those with prosthetic valve IE, right-sided native valve IE, and cardiac device infection [4]. According to the new criteria, definite IE is established if pathologic criteria, which include either microbiologic or histopathologic criteria, are met. Microbiologic criteria require that microorganisms be identified in vegetation, from cardiac tissue, explanted prosthetic valve/sewing ring, ascending aortic graft, endovascular cardiac implantable electronic device, or from an embolus [3]. Histopathologic criteria require that active endocarditis (acute, subacute, or chronic) be identified in the sources mentioned above with the microbiologic criteria [3]. Our patient met the microbiologic criteria as the explanted aortic valve vegetation was cultured and grew alpha-hemolytic *Streptococcus*. He also met the modified Duke criteria, which outlines two major criteria and five minor criteria. The diagnosis of IE can be made by the modified Duke criteria if two of the major criteria are met, or if one of the major and three of the minor criteria are met, or if all five of the minor criteria are met. The major criteria are blood cultures positive for typical microorganisms (i.e., *Staphylococcus aureus*, *Enterococcus*, *Viridans streptococcus*) consistent with IE from two separate blood cultures and echocardiogram showing endocardial involvement, whether it be valvular vegetation, abscess, or new valvular regurgitation [3]. Minor criteria include predisposing heart condition or IV drug use, fever, vascular phenomena (major arterial emboli, septic pulmonary infarcts, and Janeway’s lesions), immunologic phenomena (glomerulonephritis, Osler’s nodes, Roth’s spots, rheumatoid factor), microbiologic evidence (positive blood cultures but does not meet the major criterion described above) [3]. Symptoms of bacterial endocarditis, including fever, chills, chest pain, shortness of breath, fatigue, and joint or muscle aches, can occur within weeks to months after a dental procedure. 

*S. sanguinis *endocarditis is a unique and relatively rare diagnosis, especially after dental work. There is only one case in the current literature of *S. sanguinis* endocarditis; however, that patient's diagnosis was associated with orthodontic bracing [4]. This case is unique in that the patient had known BAV and only had a routine dental cleaning without a significant dental procedure or other comorbidities that could have led to his diagnosis. The pathophysiology of IE in BAV is hypothesized to be related to the shear stress of turbulent blood flow across bicuspid valves [2]. The shear stress leads to endothelial tissue damage, leading to platelet and fibrinogen deposition, which facilitates fibrosis and subsequent valve calcification, making it susceptible to bacterial/fungal seeding [2].

Timely surgical intervention is crucial in the treatment of IE and restoration of valve function [4]. Early valve replacement has been shown to significantly lower mortality in patients with IE [4]. Some complications of IE include heart failure secondary to valvular insufficiency, perivalvular abscess, and septic embolization leading to stroke or renal abscess/infarct [3]. The risk of embolization decreases after appropriate antibiotic therapy is initiated. This is why it is important to begin broad-spectrum empiric coverage and then narrow down antibiotics based on culture sensitivities. The duration of treatment for *S. sanguinis* IE is four to six weeks of intravenous antibiotics.

BAV is currently considered an “intermediate” risk for IE, therefore, according to current American Dental Association guidelines, antibiotic prophylaxis for these patients is technically not indicated [5]. Current guidelines state that those with prosthetic cardiac valves, prosthetic material used for cardiac valve repairs, prior history of IE, cardiac transplantation with valve regurgitation, unrepaired cyanotic congenital heart disease, and repair congenital heart defects with residual shunts or valvular regurgitation mandate antibiotic prophylaxis [5]. A systematic review published in July 2023 found that BAV patients are at 12-fold higher risk compared to patients with tricuspid aortic valve (TAV) [2]. The incidence of IE in BAV patients is 48 cases per 10,000 per year compared to 10 cases per 100,000 TAV patients per year [2]. This case signifies that even routine dental cleaning can be the source of life-threatening infection. Our patient’s outcome with his dental cleaning combined with the data from the systematic review suggest that BAV patients are at high risk of IE, emphasizing the need for prophylactic antibiotics in routine dental cleaning as well as more invasive dental procedures in those with BAV.

## Conclusions

The present case of *S. sanguinis* endocarditis in a 63-year-old male with BAV with literature review that shows that BAV patients are at higher risk compared to patients with tricuspid aortic valve emphasizes the need for prophylactic antibiotics prior to dental work in those with BAV.
